# Response to the letter to the Editor entitled “Heat Stress and Contaminated Hydration; A Dual-Edged Role in CKD of Uncertain Etiology”

**DOI:** 10.1016/j.ekir.2026.103792

**Published:** 2026-01-21

**Authors:** Pralaya Biswas, Ashish Kumar Sahu, Sourav Shristi, Iswar Baitharu

**Affiliations:** 1 Department of Environmental Sciences, Laboratory of Renal Toxicopathology, Sambalpur University, Burla, Odisha, India; 2 Biochemistry Laboratory, School of Life Sciences, Sambalpur University, Burla, Odisha, India; 3 Department of Nephrology, Veer Surendra Sai Institute of Medical Sciences and Research, Burla, Odisha, India

The author replies:

We appreciate the thoughtful comments of Bikbov *et al*. 2026,^1^ on our article on heat stress, dehydration, and their association with chronic kidney disease of uncertain (CKDu) etiology in Indian farming communities.^2^ We concur that CKDu is a multifactorial condition, and heat stress alone may not fully explain the disease burden in endemic regions. The points raised about nephrotoxic pesticide contamination in environmental (soil and water) as well as biological (serum and urine) matrices, as highlighted in our earlier work, are indeed important in broadening the understanding of CKDu etiology.^3^ Environmental exposures including pesticides, heavy metals, silica, and other contaminants likely act synergistically with occupational heat stress and recurrent dehydration to accelerate kidney injury.^4^ The suggestion that both ingestion (through contaminated drinking water) and inhalation (e.g., silica particulates from sugarcane burning) could contribute to disease pathogenesis is well taken.

We agree that the interaction between heat-induced dehydration and rehydration with unsafe water sources is a critical pathway that warrants further investigation. Such combined exposures may help explain variations in CKDu prevalence and severity across different geographic and occupational contexts.^5^ We also support the view that addressing CKDu requires an integrated and multidisciplinary approach. Interventions aimed at occupational safety, access to safe drinking water, air quality improvement, and systematic environmental monitoring are essential.^6^ These should be complemented by continued epidemiological and mechanistic studies to disentangle the complex interplay of environmental, occupational, genetic, and social determinants.

In conclusion, we appreciate the emphasis placed on expanding the scope of CKDu research beyond heat stress alone. Collaborative efforts across health, agriculture, and environmental sectors will be indispensable for effective prevention and protection of vulnerable farming communities.
